# How are patient-reported pain outcomes associated with biomarker and structural pathology subtypes in knee osteoarthritis? An explorative evaluation in the IMI-APPROACH cohort

**DOI:** 10.1016/j.ocarto.2025.100726

**Published:** 2025-12-11

**Authors:** M.P. Jansen, S.C. Mastbergen, W. Wirth, F.W. Roemer, J. Bacardit, A.C. Bay-Jensen, M. Kloppenburg, F.J. Blanco, I.K. Haugen, F. Berenbaum, N. Eijkelkamp, M. Jarraya

**Affiliations:** a Department of Rheumatology & Clinical Immunology, University Medical Center Utrecht, Utrecht University, Utrecht, the Netherlands; b Research Program for Musculoskeletal Imaging, Center for Anatomy and Cell Biology & Ludwig Boltzmann Institute for Arthritis and Rehabilitation (LBIAR), Paracelsus Medical University, Salzburg, Austria; c Chondrometrics GmbH, Freilassing, Germany; d Quantitative Imaging Center, Department of Radiology, Boston University School of Medicine, Boston, MA, USA; e Department of Radiology, Universitätsklinikum Erlangen and Friedrich-Alexander-Universität Erlangen-Nürnberg (FAU), Erlangen, Germany; f Interdisciplinary Computing and Complex BioSystems (ICOS) Research Group, School of Computing, Newcastle University, Newcastle upon Tyne, United Kingdom; g ImmunoScience, Nordic Bioscience, Herlev, Denmark; h Department of Rheumatology, Leiden University Medical Center, Leiden, the Netherlands; i Clinical Epidemiology, Leiden University Medical Center, Leiden, the Netherlands; j Grupo de Investigación de Reumatología (GIR), INIBIC – Complejo Hospitalario Universitario de A Coruña, SERGAS, Centro de Investigación CICA, Departamento de Fisioterapia y Medicina, Universidad de A Coruña, A Coruña, Spain; k Center for Treatment of Rheumatic and Musculoskeletal Diseases (REMEDY), Diakonhjemmet Hospital, Oslo, Norway; l Department of Rheumatology, AP-HP Saint-Antoine Hospital, Paris, France; m Sorbonne University, INSERM CRSA, Paris, France; n Center for Translational Immunology, University Medical Center Utrecht, Utrecht University, Utrecht, the Netherlands; o Department of Radiology, Massachusetts General Hospital, Harvard Medical School, Boston, MA, USA

**Keywords:** Pain, Phenotyping, Imaging, Neuropathic, Biomarkers

## Abstract

**Objective:**

To explore associations between patient-reported pain outcomes and knee osteoarthritis (OA) subtypes based on systemic biochemical markers and joint structural pathology as defined by MRI.

**Methods:**

Data were obtained from 297 knee OA patients from the IMI-APPROACH study. Pain outcomes were assessed using the KOOS, WOMAC, ICOAP, NRS, PainDETECT, and a pain diary. Biochemical markers in serum and urine were used to classify patients into systemic biomarker subtypes (low tissue turnover, structural damage, and systemic inflammation) via k-means clustering. Structural pathology subtypes were determined using MRI into an inflammatory, meniscus/cartilage damage, and subchondral bone pathology subtype. Associations between pain measures and subtypes were analyzed using multivariable regression models adjusted for age, sex, and BMI.

**Results:**

The systemic inflammation biomarker subtype was significantly associated with higher KOOS pain, WOMAC weight-bearing pain, NRS knee pain, and PainDETECT scores (all p ​≤ ​0.042 and β ​≥ ​0.12). The low tissue turnover subtype negatively associated with lower KOOS, WOMAC, and ICOAP constant pain (all p ​≤ ​0.22 and β ​≤ ​−0.13), and the structural damage subtype with lower PainDETECT scores (more nociceptive-like pain; p ​= ​0.046 and β ​= ​−0.12). Among MRI subtypes, meniscus/cartilage damage was significantly associated with lower PainDETECT scores (p ​= ​0.005 and β ​= ​−0.16). No significant associations were found for the subchondral bone subtype or pain diary outcomes.

**Conclusion:**

For commonly used pain questionnaires, pain severity seems linked with inflammatory activity more than structural damage. Structural damage is primarily associated with nociceptive-like pain according to PainDETECT, which might be valuable for patient selection to clinical trials and observational studies.

## 1 Introduction

Knee osteoarthritis (OA) is a highly prevalent disease characterized by structural changes such as cartilage loss, osteophyte formation, subchondral bone remodeling, and synovial inflammation. Clinically, pain is the primary reason for patients to seek medical care [1]. In knee OA research, pain is typically assessed through patient-reported questionnaires. Despite the availability of various questionnaires, establishing a consistent link between patient-reported pain and measurable systemic or local variations, such as wet biomarkers or structural pathology from imaging, remains challenging. Previous studies often focused on single markers and have found some associations between pain and features like synovitis or bone marrow lesions. However, grouping patients into subtypes, potentially reflecting pathological mechanisms, rather than examining isolated markers, may provide better insights into how different questionnaires capture systemic or local disease changes [2]. Therefore, this exploratory, hypothesis-generating study aimed to evaluate the relationship between patient-reported pain outcomes and knee OA subtypes based on serum and urine biomarkers and MRI structural pathology.

## 2 Methods

### 2.1 Patient data

Data was obtained from the IMI-APPROACH cohort study database [3]. This observational, longitudinal study enrolled 297 patients with knee OA according to ACR criteria at 5 clinical centers in Europe, meaning they had clinical knee OA with knee pain but not necessarily radiographic OA (Kellgren-Lawrence (KL) grade ≥2). The index knee was defined for each patient based on clinical knee OA criteria and pain as indicated by the patient. In case both knees were affected equally, the right knee was selected. Demographic data, questionnaires, biomarker levels from blood and urine samples, and index knee semi-quantitative MRI Osteoarthritis Knee Scores scored by an experienced radiologist were present [4].

### 2.2 Pain outcomes

Pain subscores were calculated from the Knee injury and Osteoarthritis Outcome Score (KOOS, 0–100) and Western Ontario and McMaster Universities Osteoarthritis Index (WOMAC, 0–100) questionnaires. Additionally, the three WOMAC pain questions on weight-bearing pain (pain with walking, standing, and going up or down stairs) were averaged and rescaled to obtain a composite of WOMAC weight-bearing pain (0–100), which has been used in previous studies but has not been validated [5]. From the Intermittent and Constant OsteoArthritis Pain questionnaire (ICOAP, 0–100), ICOAP constant and ICOAP intermittent pain were calculated. In addition, patients scored their index knee pain using a numeric rating scale (NRS, 0–10). Using a one-month pain diary, patients filled out each day whether they experienced pain in their index knee (yes or no), from which the percentage of days patients experienced pain was calculated. Lastly, patients completed the PainDETECT questionnaire, which evaluates the likelihood of neuropathic-like *vs* nociceptive-like pain (-1–38, with a score ≤12 indicating likely nociceptive-like pain and score ≥18 likely neuropathic-like pain) [6].

For all outcomes, a higher score represents more pain, except for PainDETECT, where a higher score indicates more neuropathic-like pain (*vs* nociceptive-like pain).

### 2.3 Biomarker subtypes

The IMI-APPROACH cohort has previously been clustered into three endotypes on the basis of serum and urine biomarkers of systemic inflammation and bone and cartilage matrix protein turnover: patients with low tissue turnover, structural damage, and systemic inflammation [7].

### 2.4 Structural pathology subtypes

Using MRI Osteoarthritis Knee Scores scores, patients were classified into three structural subtypes according to the Rapid OsteoArthritis MRI Eligibility Score (ROAMES) system: inflammatory (Hoffa- or effusion-synovitis ≥2), meniscus/cartilage (any meniscal substance loss/maceration and ipsi-compartmental cartilage damage grades ≥2.1) and subchondral bone (maximum subregional BML size of grade 3 in at least one of two knee compartments) [8]. Patients were grouped according to the presence or absence of each of these subtypes; as such it is possible for patients to have multiple ROAMES subtypes.

### 2.5 Statistical analysis

Multivariable linear regression models were used to evaluate the association between each individual different subtypes and the pain measures. Pain measures were included as the dependent variable, and the subtype as an independent binary variable (present/absent) together with age, sex and BMI. As such, the comparator group was, for every model, those without the evaluated subtype.

Since patients can exhibit more than one structural pathology subtype, sensitivity analyses were performed in case significant associations were found for a pain measure with multiple MRI pathologies. In these sensitivity analyses, patients were separated into those that only have one subtype and those that have multiple.

For all analyses, a p-value of <0.05 was considered statistically significant.

## 3 Results

### 3.1 Patients

Baseline demographics, pain outcomes and subtype distributions for the 297 patients are shown in Supplementary Table S1. Around half of patients (46 ​%) did not have radiographic OA and 77 ​% were women. Subtype presence varied from 25 ​% (MRI inflammatory subtype) to 41 ​% (MRI subchondral bone subtype).

### 3.2 Associations between systemic biomarker subtypes and pain

Patients with the systemic inflammation biomarker subtype had significantly more KOOS pain, WOMAC weight-bearing pain, and NRS pain, and more neuropathic-like pain according to PainDETECT (Table 1). Patients with the low tissue turnover subtype had significantly less KOOS pain, WOMAC pain, WOMAC weight-bearing pain, and ICOAP constant pain (Table 1). The structural damage subtype showed no association with the pain questionnaires, except for PainDETECT, where patients with structural damage biomarkers had lower PainDETECT scores suggesting more nociceptive-like pain (Table 1). The number of days patients experienced pain according to the pain diary did not show any significant association with biomarker subtypes, and neither did ICOAP intermittent pain (Table 1).

**Table 1:** Associations between biomarker subtypes and pain outcomes relevant to the index knee.

Systemic biomarker subtype		KOOS (0–100)	WOMAC (0–100)	WOMAC WB (0–100)	ICOAP (C) (0–100)	ICOAP (I) (0–100)	NRS (0–10)	Diary (0–100)	PainDETECT (-1–38)
Inflammation (n ​= ​99)	B	**5.15**	4.38	**5.00**	4.79	4.47	**0.69**	4.25	**2.08**
	β	**0.13**	0.11	**0.12**	0.11	0.10	**0.12**	0.05	**0.16**
	p	**0.024**	0.061	**0.042**	0.064	0.075	**0.038**	0.406	**0.007**
Low tissue turnover (n ​= ​96)	B	**−5.20**	**−5.29**	**−5.54**	**−5.94**	−4.27	−0.64	−4.15	−0.51
	β	**−0.13**	**−0.13**	**−0.13**	**−0.13**	−0.10	−0.11	−0.05	−0.04
	p	**0.021**	**0.022**	**0.022**	**0.020**	0.086	0.054	0.423	0.506
Structural damage (n ​= ​100)	B	−0.18	1.00	0.64	1.22	−0.11	−0.04	−0.20	**−1.51**
	β	−0.00	0.03	0.02	0.03	−0.00	−0.00	−0.00	**−0.12**
	P	0.938	0.664	0.790	0.634	0.963	0.902	0.968	**0.046**

KOOS: Knee injury and Osteoarthritis Outcome Score; WOMAC: Western Ontario and McMaster Universities Osteoarthrtis Index; WB: weight-bearing; ICOAP: Intermittent (I) and Constant (C) OsteoArthritis Pain; NRS: numeric rating scale; B: unstandardized regression coefficient; β: standardized regression coefficient. For PainDETECT, a higher score indicates more neuropathic-like pain *vs* nociceptive-like pain; for all other questionnaires, a higher score indicates more pain.

### 3.3 Associations between local structural pathology and pain

Patients with the ROAMES inflammation subtype had significantly more KOOS and ICOAP constant pain, while those with the meniscus/cartilage damage subtype additionally had significantly more nociceptive-like pain according to PainDETECT (Table 2). There were 46 patients (16 ​%) with both the inflammation and meniscus/cartilage damage subtype. When evaluating those with only the inflammation or only the meniscus/cartilage damage subtype, only the association between meniscus/cartilage damage and PainDETECT remained significant. Only patients with both the inflammation and meniscus/cartilage ROAMES subtype showed a significant association with KOOS and ICOAP constant pain. The subchondral bone subtype did not show any significant association with the pain outcomes (Table 2).

**Table 2:** Associations between MRI structural pathology subtypes and pain outcomes relevant to the index knee.

MRI structural pathology subtype		KOOS (0–100)	WOMAC (0–100)	WOMAC WB (0–100)	ICOAP (C) (0–100)	ICOAP (I) (0–100)	NRS (0–10)	Diary (0–100)	PainDETECT (-1–38)
Inflammation (n ​= ​71)	B	**6.21**	4.50	4.50	**8.58**	3.55	0.49	7.65	−1.08
	β	**0.14**	0.10	0.10	**0.17**	0.07	0.08	0.09	−0.08
	p	**0.012**	0.078	0.094	**0.002**	0.191	0.176	0.177	0.193
Meniscus/cartilage (n ​= ​104)	B	**5.86**	3.79	3.28	**6.64**	3.67	0.60	5.37	**−2.07**
	β	**0.15**	0.09	0.08	**0.15**	0.09	0.11	0.07	**−0.16**
	p	**0.008**	0.099	0.175	**0.008**	0.132	0.066	0.280	**0.005**
Subchondral bone (n ​= ​119)	B	0.84	−0.93	−0.27	0.00	0.55	0.13	−0.93	−0.75
	β	0.02	0.02	−0.01	0.00	0.01	0.02	−0.01	−0.06
	p	0.700	0.680	0.909	1.00	0.816	0.682	0.849	0.299

KOOS: Knee injury and Osteoarthritis Outcome Score; WOMAC: Western Ontario and McMaster Universities Osteoarthrtis Index; WB: weight-bearing; ICOAP: Intermittent (I) and Constant (C) OsteoArthritis Pain; NRS: numeric rating scale; B: unstandardized regression coefficient; β: standardized regression coefficient. For PainDETECT, a higher score indicates more neuropathic-like pain *vs* nociceptive-like pain; for all other questionnaires, a higher score indicates more pain.

## 4 Discussion

This study explored the associations between different pain questionnaires and systemic and local OA subtypes in knee OA patients. The findings provide insight into the relationship between biomarker subtypes, structural pathology, and patient-reported pain outcomes.

The inflammation biomarker subtype was consistently associated with higher pain scores in several questionnaires, including KOOS, WOMAC weight-bearing, and NRS. These patients also showed higher PainDETECT scores, suggesting that inflammatory activity is linked to more neuropathic-like pain. Previously, neuropathic-like pain has been associated with neuroinflammation in particular, which may explain why no significant association with ROAMES joint inflammation was found [9]. The low tissue turnover subtype was associated with lower pain scores, particularly in KOOS, WOMAC, and ICOAP constant pain, suggesting that lower tissue turnover may reflect a less painful disease state or, alternatively, an absence of inflammatory activity. Surprisingly, there was almost no association between the structural damage systemic biomarker subtype and most pain outcomes. The only significant association observed was with PainDETECT, where patients with the structural damage subtype exhibited more nociceptive-like pain. This suggests that structural degradation at the biochemical level does not necessarily translate into increased pain perception, except in relation to categorization of pain and potentially to its underlying mechanisms.

Among the MRI-based structural pathology subtypes, the KOOS pain and ICOAP constant pain might reflect a combination of local inflammation and meniscus/cartilage damage groups. This suggests that the combination of these two MRI-defined subtypes captures important aspects of painful OA pathology. The meniscus/cartilage subtype was also significantly associated with lower PainDETECT scores, indicating that structural damage in these regions is linked to nociceptive-like pain. These findings confirm previous results, where comparing patients with neuropathic-like pain with matched on knee pain level with patients with nociceptive-like pain showed that neuropathic patients had significantly less joint damage despite having similar levels of pain [6]. Unexpectedly, the subchondral bone subtype was not associated with any pain outcomes. This is surprising given prior studies linking bone marrow lesions with pain in knee OA, although these associations are frequently inconsistent between studies [10]. A previous study using the same structural phenotyping criteria did find a significant association between the subchondral bone subtype and subsequent radiographic and pain progression [8]. This could be because that study evaluated longitudinal progression instead of cross-sectional associations, or because different inclusion criteria, as the study only included KL grade ≥2 patients. However, the authors did not find a significant association when evaluating pain progression alone, indicating the radiographic progression may have been driving the significant findings.

Both the pain diary and the ICOAP intermittent pain did not show any significant associations with systemic biomarker or local pathology subtypes, although the ICOAP intermittent pain indicated at least some trend towards higher pain levels in inflammatory patients. A previous study in knee OA patients similarly found that constant pain more than intermittent pain was associated with synovitis as assessed with ultrasound [11]. Both the pain diary and ICOAP intermittent pain scale in some way reflect a fluctuation in pain as opposed to constant pain levels. According to these results, fluctuations in pain may not be related to knee OA disease activity, and (constant) pain levels might be more relevant. In IMI-APPROACH, the pain diary evaluated only whether the patient experienced pain on each day, expressed here as a percentage of days with pain. It did not include questions about the level of pain, which might have provided more insight about pain fluctuation [12].

The results of this study suggest that commonly used pain questionnaires may be more related to inflammatory activity than local structural pathology in knee OA. Some measures – particularly KOOS pain, ICOAP constant pain, and to a lesser extent WOMAC weight-bearing pain – appear to be reflective of inflammatory processes and could serve as indicators of inflammatory pain. Synovitis is one of the few structural markers that has been linked to pain in knee OA [13]. The PainDETECT questionnaire demonstrated associations with both systemic biomarkers and local pathology, suggesting that it may have potential utility for patient stratification. Specifically, patients with nociceptive-like pain may exhibit more structural damage and might be of more interest in inclusion in disease-modifying trials where the desired effect is an improvement in both structure and symptoms. On the other hand, those with neuropathic-like pain may have more systemic and local inflammatory activity and might be a more interesting group of patients when evaluating anti-inflammatory treatments. Indeed, a placebo-controlled randomized controlled trial evaluating the effects of an oral anti-inflammatory drug showed significant symptomatic improvement only in patients with a baseline PainDETECT score >12 indicating non-nociceptive pain [14]. Importantly, for all associations, β values were generally low. As such, despite statistical significance, the subtypes only explain a small part of the variation in pain. Additionally, B-values were low as well. For example, B-values for significant KOOS associations were between 5 and 7, while the minimal clinically important difference of the KOOS pain is 15.4 [15].

This study has limitations. First, because of the cross-sectional design, no conclusions can be drawn concerning causality between subtypes and pain measures. Longitudinal analyses are needed to assess how these associations evolve over time and whether there might be predictive value for disease progression and treatment response. Second, due to the exploratory nature, a broad set of biomarkers and imaging features were analyzed, without correcting for multiple testing. Which associations would remain significant would depend on the chosen methods of multiple testing correction. For example, applying Benjamini–Hochberg false discovery rate correction would leave all currently significant associations statistically significant at q ​= ​0.20 (exploratory) and all but three (those with p ​≥ ​0.038 in Table 1) at q ​= ​0.10 (stricter). The fact that different types of pain and subtype evaluation show high agreement indicates that multiple testing may not have been an issue, but follow-up studies should evaluate and validate findings in this study with specific hypotheses. Third, while all included patients had clinical knee OA, only around half had radiographic OA (KL ​≥ ​2). This could have influenced results, as those without radiographic OA might also have lower biomarker levels and MRI damage, and future studies with a larger number of patients might allow for analyses in those with (and without) radiographic OA separately.

In conclusion, this study provides evidence of associations between subtypes of knee OA liquid biomarkers, joint tissue pathology, and pain. The most frequently used questionnaires in knee OA, namely the KOOS and WOMAC, seem more reflective of local and systemic inflammatory activity than structural tissue damage. The PainDETECT is significantly associated with both systemic markers and local pathology and might be useful for patient selection. These findings offer clues into the long-standing challenge of connecting the different disease domains in knee OA and could improve diagnostic tools and patient selection.

## Author contribution

M.P. Jansen: conceptualization, methodology, formal analysis, investigation, writing – original draft, project administration.

S.C. Mastbergen: methodology, resources, writing – review & editing, supervision, funding acquisition.

W. Wirth: conceptualization, resources, writing – review & editing.

F.W. Roemer: formal analysis, resources, writing – review & editing.

J. Bacardit: formal analysis, methodology, writing – review & editing.

A.C. Bay-Jensen: formal analysis, resources, writing – review & editing.

M. Kloppenburg: resources, writing – review & editing, funding acquisition.

F.J. Blanco: resources, writing – review & editing, funding acquisition.

I.K. Haugen: resources, writing – review & editing, funding acquisition.

F. Berenbaum: resources, writing – review & editing, funding acquisition.

N. Eijkelkamp: methodology, supervision, writing – review & editing.

M. Jarraya: conceptualization, methodology, formal analysis, writing – review & editing.

## Role of the funding source

The sponsors had no role in the study design, collection, analysis and interpretation of data, in the writing of the manuscript, or in the decision to submit the manuscript for publication.

## Conflicts of interests

WW: Employee and shareholder of Chondrometrics GmbH.

FWR: Shareholder of Boston Imaging Core Lab, LLC and consultant to Grünenthal GmbH. Editor in Chief Osteoarthritis Imaging.

MK: Consulting fees from Pfizer, CHDR, Novartis, UCB, GSK, and Peptinov, all paid to institution. Royalties from Wolters-Kluwer and Springer Verlag, all paid to institution.

FJB: Funding from Gedeon Richter Plc., Bristol-Myers Squibb International Corporation, Sun Pharma Global FZE, Celgene Corporation, Janssen Cilag International N.V, Janssen Research & Development, Viela Bio, Inc., Astrazeneca AB, UCB BIOSCIENCES GMBH, UCB BIOPHARMA SPRL, AbbVie Deutschland GmbH & Co.KG, Merck KGaA, Amgen, Inc., Novartis Farmacéutica, S.A., Boehringer Ingelheim España, S.A, CSL Behring, LLC, Glaxosmithkline Research & Development Limited, Pfizer Inc, Lilly S.A., Corbus Pharmaceuticals Inc., Biohope Scientific Solutions for Human Health S.L., Centrexion Therapeutics Corp., Sanofi, TEDEC-MEIJI FARMA S.A., KiniksaPharmaceuticals, Ltd. Grunenthal.

IKH: Research grant (ADVANCE) from Pfizer (payment to institution) and personal fees from Abbvie, Novartis, Grünenthal and GSK, outside of the submitted work.

FB: consulting fees from Grunenthal, GSK, Eli Lilly, Novartis, Pfizer, Servier, and 4P Pharma; honoraria from Viatris, Pfizer, and Zoetis; travel support from Nordic Pharma; holds patents related to 4Moving Biotech; serves on advisory boards for AstraZeneca, Sun Pharma, and Nordic Bioscience; and holds stock in 4P Pharma and 4Moving Biotech. CMO and co-founder of 4Moving Biotech.

The other authors report no conflict of interest.
