# Supplementary material for: How are patient-reported pain outcomes associated with biomarker and structural pathology subtypes in knee osteoarthritis? An explorative evaluation in the IMI-APPROACH cohort
Source: Osteoarthr Cartil Open. 2025 Dec 11;8(1):100726. doi: 10.1016/j.ocarto.2025.100726 (PMC12794487; doi:10.1016/j.ocarto.2025.100726)
Supplement: Multimedia component 1 [file mmc1.docx]

**Supplementary data**

Supplementary Table S1: Cohort overview.

| **Parameter** | **Patients**  **Mean ± SD or n (%)** |
| --- | --- |
| Age, years | 66.5 ± 7.1 |
| BMI, kg/m^2^ | 28.1 ± 5.3 |
| Female sex | 230 (77) |
| KL grade   - 0 - 1 - 2 - 3 - 4 | 56 (19)  79 (27)  64 (22)  87 (29)  11 (4) |
| *Pain measures relevant to index knee* | |
| KOOS pain (0-100) | 33.9 ± 18.9 |
| WOMAC pain (0-100) | 30.3 ± 19.2 |
| WOMAC weight-bearing pain (0-100) | 33.8 ± 20.3 |
| ICOAP knee constant (0-100) | 22.7 ± 21.6 |
| ICOAP knee intermittent (0-100) | 31.5 ± 20.9 |
| NRS index knee (0-10) | 4.6 ± 2.7 |
| Pain diary (0-100) | 60.0 ± 37.5 |
| PainDETECT (-1–38) | 9.1 ± 6.2 |
| *Pain measures specific to other body parts* | |
| NRS contralateral knee (0-10) | 3.0 ± 2.6 |
| NRS index hip (0-10) | 2.4 ± 2.8 |
| NRS contralateral hip (0-10) | 2.0 ± 2.6 |
| NRS lower back (0-10) | 4.2 ± 3.2 |
| NRS hands (0-10) | 3.4 ± 2.8 |
| HOOS pain (0-100) | 19.1 ± 20.8 |
| ICOAP hip constant (0-100) | 13.2 ± 19.0 |
| ICOAP hip intermittent (0-100) | 18.8 ± 21.1 |
| *Systemic biomarker subtypes* | |
| Inflammation | 99 (34) |
| Low tissue turnover | 96 (33) |
| Structural damage | 100 (34) |
| *MRI structural pathology subtypes* | |
| Inflammatory | 71 (25) |
| Meniscus/cartilage damage | 104 (36) |
| Subchondral bone | 119 (41) |
